# Advanced Wearable Sensors Technologies for Healthcare Monitoring

**DOI:** 10.3390/s25020322

**Published:** 2025-01-08

**Authors:** Toshiyo Tamura

**Affiliations:** Future Robotics Organization, Waseda University, Tokyo 169-8050, Japan; t.tamura3@kurenai.waseda.jp or t.tamura1949@gmail.com

## 1. Introduction

Wearable sensor technologies are rapidly evolving and expanding their reach into critical wellness and healthcare applications. This progress is driven by advances in sensing, computing, wireless communication, signal processing, and pattern recognition. Wearable devices can collect a variety of physiological information. Human motion analysis began in the 1990s with advanced high-performance, compact accelerometers. Quantitative analysis became possible, and research in the fields of gait and rehabilitation progressed rapidly. Subsequently, the wearable sensor was applied not only in the field of motion analysis [1,2] but also in the fields of cardiovascular system, metabolism, and body temperature. In particular, pulse rate monitors using green LEDs are widely used. Various physical, chemical, and biological sensors have been produced to collect physiological information in real-time and in a non-invasive or minimally invasive manner. What is special about wearable sensors is that they are less expensive and easier to use than other systems [3].

Wearable technologies facilitate the extension of monitoring into the community and have been used in numerous clinical applications, including the monitoring of healthy, elderly, and frail individuals, individuals with neurological disorders, measurement of physical activity in disease association studies, and the development of behavioral interventions [4]. In clinical practice, wearable devices have been evaluated to assess individuals with Parkinson’s disease [5], hypertension [6], and diabetes [7].

The goal of this Special Issue is to highlight state-of-the-art applications of wearable sensors with a focus on the wellness and healthcare applications of the technology.

## 2. Contributed Papers

The following Special Issue includes ten contributions. Five of the ten papers focus on motion and force measurements, followed by respiratory function, ECG signals, cardiovascular (blood pressure), body temperature, and sweat measurements.

Contribution 1: To predict fall risk, Dotov et al. developed a clinical screening test with motion sonification and inertial motion sensors to accurately measure and manipulate the interaction between stimuli in the environment and the entire body. Participants’ hand movements were sonicated to synchronize stimulus and hand movements. Supra-postural coordination leads to postural control. The results showed that a decrease in super-postural coordination decreases the ability to decouple postural control from sensory–motor and cognitive activities.

Contribution 2: The goal of this paper is to assess the validity of the Ergotex Inertial Measurement Unit (IMU) against a 3D motion capture system for measuring trunk, hip, and shoulder angles. The results showed that the Ergotex IMU is a reliable tool for accurate joint angle measurements.

Contribution 3: The authors of this paper present a investigation on a non-intrusive technique to evaluate the fit of orthopedic prosthesis sockets in transfemoral amputees based on experimentally obtained vibrational data. Based on the experimental investigations shown and the derived results, it can be concluded that structural dynamic measurements are a promising non-intrusive technique to evaluate the fit of orthopedic prosthesis sockets in transfemoral amputee patients.

Contribution 4: The authors of this paper propose a method to assess gait disturbance by observing changes in the medial–lateral center of pressure (COP) during the gait cycle; the authors proposed polar coordinate gaitgrams and two indices, namely the area ratio index and the slope of the tangent line common to the two closed curves. Using these indices, they experimentally verified the differentiation between stroke patients and healthy subjects. The results showed that the proposed polar coordinates and indices could be used to develop and apply a portable device to evaluate gait disorders.

Contribution 5: Lin et al. developed self-adhesive, stretchable fabric, nanocomposite skin strain sensors to collect information on muscle contraction during exercise and then validated them for human exercise monitoring. The developed sensor was compared with optical motion capture by the participants. The results confirmed that the sensor could reliably measure tensile and compressive skin strain across the calf and tibialis anterior muscles. In addition, the response of the elastic fabric sensors correlated well with sEMG muscle engagement measurements. This sensor is also free of motion artifacts commonly observed when using sEMG in a free-living community environment.

Contribution 6: Accurate tidal volume measurements based on respiration-induced upper body surface motion could be valuable in clinical and sports monitoring applications. The authors of this paper evaluate the theoretical ability of different sensors to measure tidal volume. The highest correlation was found between spirometer volume and upper body circumference. Surface deflection also showed a strong correlation. Several thoracic kinematic parameters that can be measured with common sensors and their correlation with tidal volume are presented in this study.

Contribution 7: Noise is a frequently encountered issue in portable electrocardiogram (ECG) monitoring systems. An et al. addressed motion artifacts using adaptive filtering, a specially designed ECG device, and an impedance pneumography (IP) data acquisition system. Subjective and objective evaluation of the performance of the proposed motion artifact reduction method proved that the method could suppress motion artifacts and minimize distortion of the denoised ECG signal.

Contribution 8: The device developed in this study is not wearable but is an unobtrusive device. The principle presents a radar-based approach that extracts pressure waves using skin movement caused by arterial pulsation. The skin movements were used as input for a neural network-based regression model. The trained model unfortunately did not meet the requirements of AAMI and BHS blood pressure metrics.

Contribution 9: Heat stroke is a leading issue in the context of climate change. Measuring body temperature to prevent heat stroke is important, and Tamura et al. developed a system that continuously measures tissue temperature close to the core temperature and prevents heat stroke. Based on the principle of the dual heat flow method, the system accurately measures body temperature regardless of fluctuations in environmental temperature and estimates the increase in body temperature based on temperature change.

Contribution 10: In their study, Ibrahim et al. review current wearable sweat-sensing devices. They discuss the limitations of the devices and suggest model designs, features, performance, and device operation for continuous and non-continuous flow sweat analysis. In addition, they present various sweat biomarkers that have been explored to broaden the use of non-invasive sweat samples in healthcare and related applications.

## 3. Conclusions

Several new concepts are presented in this Special Issue. The majority of the presented research is basic and requires further improvement for healthcare and clinical applications. Future directions include the Internet of Things, the Internet of Health Things, and the Internet of Medical Things becoming increasingly popular and researchers developing novel sensors in addition to related software to analyze such data. Machine learning and deep learning can be used to clarify these data. In addition, a more accurate assessment of accuracy and validity is required to maintain accuracy equivalent to that of medical devices [8]. Wearable devices are low-cost and relatively easy to operate; thus, we can expect to see increasing publication of promising research results.
